# Dental Education Once More

**Published:** 1884-09

**Authors:** 


### DENTAL EDUCATION ONCE MORE.

The three articles in the August number of the INDEPENDENT PRACTITIONER, as well as the one of the June number, are so thoroughly disingenuous that it is a difficult matter for men whose desire and practice it is to act openly and honestly to answer them properly.

The authors of those articles do not or will not understand the plain statement made by us in the July number of that journal. The character of the charges contained in the first article in the June number, and reiterated directly or indirectly in the August number of the INDEPENDENT PRACTITIONER, has been vague. The most specific charge was contained in the first article by Dr. Hopkinson, as follows: "Certain irregularities practiced by the 'present faculty,' in direct opposition to the law as it appears in her charter, and according to the terms of graduation published in the forty-fourth annual catalogue."

As to this we need only quote the following provisions of the charter, a copy of which in our possession is certified to as correct by Dr. F. J. S. Gorgas, as also is the fact that the charter was granted in 1840 for thirty years, and in 1870 renewed by the State of Maryland for another thirty years.

"Sec. 8. *And be it enacted*, that R. S. Stewart" (and others named) "be appointed a board of visitors, to be styled the Board of Visitors of the Baltimore College of Dental Surgery, who shall be empowered to examine into the state and condition of the Institution, and see that the requirements of this charter are fulfilled, five constituting a quorum; they shall meet at least once in each year, to fill vacancies that may occur in their body, appoint such officers as may be necessary to the discharge of their own duties, and attend to any other business connected with their office as visitors and supervisors of the Institution.

"Sec. 9. *And be it enacted,* that the said Professors" (provided for in the previous section) "shall have full power to confer on any student who shall have attended all the lectures in said college for two terms, and others, after an examination by the Professors [who] shall have been found worthy the degree of Doctor of Dental Surgery, and the said Professors shall have power and are hereby directed to accept evidence from any student of his having attended lectures in any respectable dental or medical school for an equal period of time, and receive the same as equivalent to his having attended one of the terms herein mentioned."

The practice of graduation on examination alone, in accordance with Section 9, "has not been confined to the last fifteen years, but has extended over a period commencing with the organization of the school." (See Gorgas, p. 418.)

Verily, the young man, to use his own language, "comes forward" too much, and "stands back" too little. Especially does he do so when he names the case of "George Horatio Jones, of England, who received his diploma, so says 'dame rumor,' by proxy, and, of course, *in absentia.*" Why, bless his ignorant soul, the delivery and reception of a diploma by proxy, which is done under certain circumstances by all educational institutions, is *not* granting the degree *in absentia;* and even if it were, that whole case was managed and pushed through by Dr. Gorgas, then Dean of the Baltimore College of Dental Surgery.

We have now before us papers in Dr. Gorgas' handwriting, and signed by him, dated April 27, 1882, in which is the following:—

"RECEIPTS."

Students since March 15, 1882.

From . . . . . .

" . . . . . .

" . . . . . .

" . . . . . .

" George H. Jones (Englishman), $135.00.

This sum is made up of the matriculation fee, five dollars; the tuition fee, one hundred dollars; and the diploma fee, thirty dollars. The present Dean was the only one of the faculty who opposed the graduation of G. H. Jones, and when elected to succeed Dr. Gorgas could do no less than carry out his contracts.

And this brings Dr. Gorgas on the stage. He says "the author of the reply must have had a vivid imagination when he inferred as the facts of these cases could only have been known to the Dean of the Dental Department of the University of Maryland, hence the latter had some part in bringing the charges. For his enlightenment it is only necessary to state that the facts of these cases were known throughout the entire country long before Dr. Hopkinson's article appeared."

And pray, from what other source than the Dean, who alone was originally in possession of these facts, could this knowledge have come both to Dr. Hopkinson and "the entire country?" The statements of Dr. Gorgas and Dr. Hopkinson could not be so nearly the same if they had not all come from the same source.

And he talks about the "altogether unprovoked attack" on the Dental Department of the University of Maryland, when he knows that the two dental members of the Faculty of that Department have for more than two years been circulating falsehoods about the Baltimore College of Dental Surgery.

Denying that our "reply" was "abusive of the University," we pronounce his "simple statement of facts," pp. 416–420, to be a tissue of misstatements, as may be proven by his own correspondence and that of Dr. J. G. Palmer, as well as by the testimony of the gentlemen whose names have been so improperly and wrongfully brought before the public, and who, as we believe, are gentlemen of honor and veracity. With this belief we will leave the questions of personal veracity to be settled by those interested, promising to give all the aid in our power to sustain the truth.

As to Dr. Harris and his article, we dismiss both in much shorter order than he was dismissed from the faculty of the Baltimore College of Dental Surgery, and for the same reasons. We simply tell him to head the affidavit which we published in the July number of the INDEPENDENT PRACTITIONER with "Clinton, Sampson Co., North Carolina," and fill the blank at the end with the name "Frank Boyette," and he will have both name and locality of the affiant.

The Maryland Dental College did not die in 1879, as stated by Dr. Hopkinson in his article, and by Dr. Gorgas in letters of his which we have, any more than a woman who marries a man and takes his name dies. The two colleges were consolidated; they were married under a contract signed by their faculties, which contract is open to the inspection of the profession and the public, and which utterly disproves their assertion. At the time of this consolidation the classes in the two colleges were nearly equal, and from that time to this the classes in the Baltimore College of Dental Surgery have been about double what they were before.

But all these publications, charges, replies, replications, rejoinders and sur-rejoinders, besides being interminable, do not settle nor decide anything. Therefore we have a proposition to make to the Dean and Professors of the Dental Department of the University of Maryland, which is, that we refer this whole matter to the officers of the State Dental Societies of New York, Pennsylvania, and New Jersey, or to the Boards of Dental Examiners of those States, who shall appoint from themselves or from the profession in those States a committee of three, or five, to come to Baltimore and hear all the testimony in the matter, giving both sides a fair and impartial hearing. That this committee shall publish the testimony on both sides, with their conclusions thereon, in the INDEPENDENT PRACTITIONER and other journals, or in such other manner as they may deem best, and that the expenses of that committee and of their publications be divided equally between and paid by the Baltimore College of Dental Surgery and the Dental Department of the University of Maryland.

James B. Hodgkin, D. D. S.,
Richard B. Winder, M. D., D. D. S.,
M. Whilldin Foster, M. D., D. D. S.,
J. H. Coyle, D. D. S.,
Thomas S. Latimer, M. D.,
James E. Lindsay, M. D.,
Governing Faculty of the Baltimore College of Dental Surgery.

